# The effects of blood flow restriction combined with resistance training on lower limb strength, muscle hypertrophy, jumping ability, and sprint speed in athletes: a systematic review and meta-analysis

**DOI:** 10.3389/fphys.2025.1612685

**Published:** 2025-07-29

**Authors:** Beiwang Deng, Gesheng Lin, Yuer Shi, Duanying Li, Zeyun Guan, Chaoming Liang, Jian Sun

**Affiliations:** ^1^ School of Athletic Training, Guangzhou Sport University, Guangzhou, China; ^2^ Guangdong Provincial Key Laboratory of Human Sports Performance Science, Guangzhou Sport University, Guangzhou, Guangdong, China; ^3^ Department of Physical Education, Guangzhou Sport University, Guangzhou, China; ^4^ Faculty of Health Sciences and Sports, Macao Polytechnic University, Macao, China; ^5^ Badminton Technical and Tactical Analysis and Diagnostic Laboratory, Guangzhou Sport University, Guangzhou, China

**Keywords:** athletes, resistance training, lower limb strength, blood flow restriction training, athletic performance

## Abstract

**Objective:**

This meta-analysis aims to evaluate the comparative effects of blood flow restriction resistance training (BFR-RT) versus traditional resistance training (RT) on lower limb muscle hypertrophy, maximal strength, jumping ability, and sprint performance in athletes.

**Methods:**

A comprehensive search was conducted across PubMed, Web of Science, the Cochrane Library, Embase and SPORTDiscus databases. This search identified 181 studies, of which 15 met the inclusion criteria. The quality of the studies was assessed using the Cochrane risk-of-bias tool, and data were analyzed using StataMP 17.0.

**Results:**

The analysis revealed that BFR-RT significantly enhanced lower limb maximal strength (ES = 0.27, 95% CI: 0.03–0.52, p = 0.031, I^2^ = 25%), demonstrating its effectiveness in improving strength. However, no significant differences were observed between BFR-RT and RT for lower limb muscle hypertrophy (ES = 0.17, 95% CI: -0.15–0.50, p = 0.293, I^2^ = 0%), jumping ability (ES = 0.25, 95% CI: -0.04 to 0.54, p = 0.091, I^2^ = 0%), or sprint performance (ES = −0.1, 95% CI: 0.39–0.19, p = 0.136, I^2^ = 0%).

**Conclusion:**

The findings suggest that while BFR-RT is effective in improving maximal strength, it does not offer additional benefits over traditional RT in terms of muscle hypertrophy, jumping ability, or sprint performance.

## Graphical Abstract

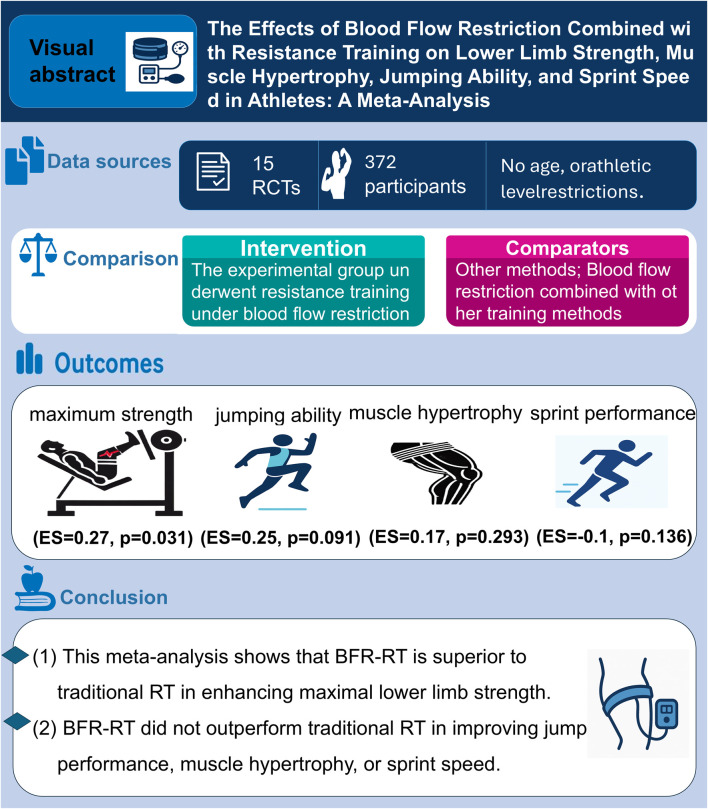

## 1 Introduction

Blood Flow Restriction Training (BFRT) is a technique that uses a cuff or tourniquet applied to the proximal limb to partially occlude blood flow during exercise (Anderson et al., 2019; Sato, 2005). By restricting venous return while allowing partial arterial inflow, BFRT creates a localized hypoxic environment that intensifies fatigue and stimulates muscular and metabolic adaptations even at low training loads (Yang et al., 2024; The Occlusion Cuff ®, 2025). This method has become increasingly widespread in both sports performance and rehabilitation contexts, as clinicians and coaches integrate BFRT to promote muscle growth, preserve function, and facilitate recovery in post-operative and athletic populations (Loenneke et al., 2015; Saraf et al., 2022). Notably, combining low-intensity exercise with vascular occlusion enables athletes to achieve gains in muscle size and strength typically associated with high-load resistance training (HL-RT), but with substantially reduced mechanical stress on joints and connective tissues (Yang et al., 2024; Lorenz et al., 2021).

Among its reported benefits, BFRT combined with resistance exercise (BFR-RT) has demonstrated considerable efficacy in eliciting hypertrophy and strength improvements comparable to conventional heavy lifting (Yang et al., 2024; Chang et al., 2024). Research indicates that BFR-RT activates similar molecular pathways, including mTOR signaling and myokine release, thereby driving protein synthesis and muscle adaptation (Yang et al., 2024). Because BFR-RT typically employs loads of only 20%–40% of one-repetition maximum (1RM), it offers a lower-risk training strategy for both healthy athletes and those recovering from injury (Lorenz et al., 2021). Additionally, BFRT has been linked to improvements in aerobic capacity and endurance performance, with studies showing greater increases in VO_2_max and time-to-exhaustion when low-load exercise is performed under vascular restriction compared to non-occluded training (Cognetti et al., 2022). From a clinical standpoint, BFRT is also used to attenuate muscle atrophy during periods of immobilization or rehabilitation (Saraf et al., 2022). Lorenz et al. and others have highlighted that BFRT enables patients to maintain strength and muscle mass when heavy loading is not yet feasible, effectively bridging early and later rehabilitation stages (Lorenz et al., 2021).

In athletic settings, BFR-RT has attracted attention as a tool to support or complement high-load training. For example, meta-analyses and controlled trials report that BFR-RT can improve explosive capabilities, such as jump height and sprint performance, and contribute to better overall conditioning (Yang et al., 2024; Wang et al., 2023). Its advantages include the use of much lighter loads (20%–40% 1RM with high repetitions) compared to traditional strength training protocols (70%–90% 1RM), which can reduce cumulative joint stress and the risk of overuse injury (Weaver et al., 2024). Despite the reduced mechanical tension, BFRT tends to induce comparable hypertrophy and strength gains while potentially allowing more frequent training sessions and less delayed-onset muscle soreness (Lorenz et al., 2021; Rodríguez et al., 2024).

Nevertheless, the precise impact of BFRT on performance-related outcomes remains subject to debate. Some studies have reported that low-load BFR-RT produces smaller increases in strength compared to traditional high-load protocols. For example, a recent meta-analysis by Chang et al. (2024) observed that while muscle hypertrophy did not differ significantly between BFR-RT and HL-RT, strength gains were modestly inferior in BFR-RT groups (Chang et al., 2024). Similarly, trials involving semi-professional soccer players found no additional improvements in sprinting or jumping ability when BFR-RT was added to regular training (Scott et al., 2017). These results suggest that in already well-trained athletes, the translation of BFR-induced adaptations to sport-specific performance may be inconsistent. In contrast, other systematic reviews and meta-analyses have highlighted substantial benefits of BFR-RT, reporting significant improvements in strength, power, speed, and endurance relative to conventional training (Wang et al., 2023). Such discrepancies likely arise from variations in training volume, frequency, cuff pressure, exercise selection, and the training status of participants.

While previous reviews have examined the effects of BFRT across diverse populations and training modalities—including aerobic BFR, passive BFR, and mixed interventions (Yang et al., 2024; Rui et al., 2023)—few studies have specifically focused on the impact of combining BFRT with resistance training in athletes. Given that BFR-RT has become the most common and practically applied form of BFRT in sports settings, there is an urgent need to clarify its relative effects on key performance indicators essential for athletic development, including lower-limb strength, muscle hypertrophy, jumping performance, and sprint speed. This study aims to concentrate on resistance training and further elucidate the influence of BFR-RT on athletic performance. Through a meta-analysis, the study will systematically evaluate the effects of BFR-RT on performance outcomes, synthesize the existing body of evidence, and determine its actual effectiveness.

## 2 Research methods

### 2.1 Search strategy

The literature search was conducted across the PubMed, Web of Science, the Cochrane Library, Embase and SPORTDiscus databases using the following keywords: “Blood flow Restriction,” “Kaatsu,” “Ischemic Training,” “BFRT Therapy,” “BFRT Therapies,” “BFRT,” “Blood Flow Restriction Training,” “Blood Flow Restriction Exercise,” “resistance training,” “resistance exercise,” “Resistance,” “Strength Training,” “Strengthening Programs,” “Weight Bearing Exercise Program,” “players,” “sportsman,” “athlete,” “sports person,” and “sportswomen.” The search timeframe covered all publications from the inception of each database until June 29, 2025. The detailed search strategy for each database is provided in Supplementary Appendix 1.

To ensure the accuracy of the literature search, two researchers independently verified the search terms. In cases where there was disagreement between the two researchers (B.D and R. Y) regarding the selection of search terms, a third researcher (G.L) made the final decision.

### 2.2 Study selection

This meta-analysis was conducted in accordance with the guidelines of the Cochrane Collaboration (Cumpston et al., 2019) and the Preferred Reporting Items for Systematic Reviews and Meta-Analyses (PRISMA) (Moher et al., 2009). The study protocol was registered with the International Platform of Registered Systematic Review and Meta-Analysis Protocols (INPLASY202480005). The Population, Intervention, Comparator, Outcomes, and Study Design (PICOS) criteria were used to define the inclusion and exclusion criteria for this meta-analysis, as outlined in Table 1.

**TABLE 1:** Inclusion and exclusion criteria for literature.

Principle	Inclusion criteria	Exclusion criteria
P	Athletes of any age or gender, without injury, illness, or other clinical conditions	Athletes with injuries, illnesses, or other clinical symptoms
I	The experimental group underwent resistance training under blood flow restriction	Other methods; Blood flow restriction combined with other training methods
C	The control group underwent resistance training	Non resistance training
O	At least one of the following outcome indicators in athletes: lower-limb strength, muscle hypertrophy, jumping performance, or sprint speed	Outcomes unrelated to these indicators or studies unable to report mean and standard deviation before and after the intervention
S	Randomized controlled trial	Non randomized controlled trials
Other	—	Conference papers, review literature, book chapters and reviews; Unable to obtain the full text; Non sports science literature

The retrieved records from each database were imported into EndNote X9 software for duplicate removal. Two researchers independently conducted an initial screening of the titles and abstracts to identify potentially relevant studies. Following this, both researchers thoroughly reviewed the full texts and assessed the studies based on the population, intervention, comparator, outcomes, and study design criteria to determine their eligibility according to the inclusion and exclusion standards. The study selection process was conducted independently by two researchers. In cases of disagreement, a third researcher (G.L) was consulted, and any conflicts were resolved through discussion until a consensus was reached. Inter-rater agreement for study eligibility assessment based on full-text review was substantial (Almost Perfect, Cohen’s kappa = 0.85).

### 2.3 Data extraction

Upon completing the search, detailed information was collected from the eligible articles. Two researchers (B.D and R. Y) independently extracted the data into a Microsoft Excel spreadsheet. The extracted data included the article title, publication year, author names, characteristics of the subjects (age, sample size of the experimental and control groups), training protocols (intervention duration, intervention methods, training frequency, training load, BFR-RT duration, cuff location, width, pressure, and intervention methods in both the experimental and control groups), outcome measures, and authors’ conclusions. If discrepancies arose in the data extracted by the two researchers, a third researcher (G.L) would extract the data and confirm the final version. Pre- and post-intervention data were recorded as mean ± standard deviation in the Excel spreadsheet, If the full text or specific data from the study were not accessible, the corresponding author was contacted to obtain the relevant information. Inter-rater reliability for study selection was calculated using Cohen’s kappa statistic (Almost Perfect, Cohen’s kappa = 0.83).

### 2.4 Risk of Bias Assessment

The quality of all included studies was assessed using the Cochrane Risk of Bias Tool. The evaluation criteria included “Low risk,” “Unclear risk,” and “High risk,” and the final results served as the basis for assessing the quality of the included studies. The quality assessment for all studies included in this meta-analysis was independently conducted by two researchers (B.D and R. Y). Inter-rater reliability for the Risk of Bias Assessment was also calculated using Cohen’s kappa statistic (Substantial, Cohen’s Kappa = 0.72).

### 2.5 Data analysis

All statistical analyses were performed using StataMP 17.0. As all variables were continuous, outcomes were reported as means and standard deviations. Standardized mean differences (SMDs) were calculated to estimate effect sizes, and forest plots were generated accordingly. A random-effects model was applied to compute pooled effect sizes across studies, regardless of heterogeneity, to account for potential clinical and methodological differences among included studies (Borenstein et al., 2021; Chandler et al., 2019). Heterogeneity was assessed using the I^2^ statistic, with values interpreted as follows: <25% indicating low heterogeneity, 25%–75% moderate, and >75% substantial heterogeneity (Van Tulder et al., 2003). When I^2^ exceeded 75%, sensitivity analyses were conducted to assess the robustness of the pooled results (Higgins et al., 2003). The magnitude of effect sizes was interpreted according to the scale proposed by Hopkins et al. 2009: <0.2 = trivial; 0.2–0.6 = small; 0.6–1.2 = moderate; 1.2–2.0 = large; 2.0–4.0 = very large; and >4.0 = extremely large. To assess potential publication bias, a funnel plot was constructed to visually inspect asymmetry in the distribution of study effect sizes. In addition, Egger’s regression test was performed to statistically evaluate the presence of publication bias (Sedgwick and Marston, 2015). A p-value of <0.05 was considered statistically significant.

## 3 Results

### 3.1 Literature search results

**FIGURE 1:**
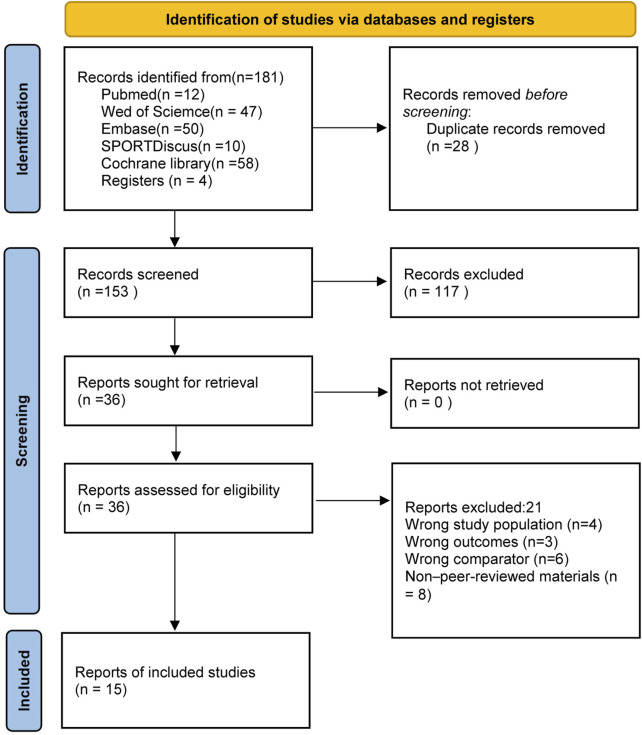
Literature screening process diagram.

A total of 181 articles were retrieved from the databases, and an additional four articles were identified through manual search of the references in related studies. After removing duplicates, an initial screening was conducted based on the titles and abstracts. Full texts were then reviewed and re-screened, excluding studies that did not meet the inclusion criteria. Ultimately, 15 studies were included in the meta-analysis. The detailed process is illustrated in Figure 1.

### 3.2 Study characteristics

A total of 15 studies were included in this meta-analysis, encompassing 372 participants, all of whom were athletes. The experimental group, which underwent BFR-RT, included 185 participants, while the control group, which performed traditional RT, included 205 participants. The majority of the interventions were conducted at a frequency of 2–3 sessions per week, with intervention durations ranging from 3 to 10 weeks. The types of athletes included in the studies were volleyball players (Wang et al., 2022), long jumpers (Hopkins et al. 2009), weightlifters (Van Tulder et al., 2003), rugby players (Jessee et al., 2018; Geng et al., 2024; Vergara et al., 2024; Adhitya et al., 2025), football players (Wang et al., 2022; Garber et al., 2011; Sarfabadi et al., 2023; Luebbers et al., 2019; Kami̇Ş et al., 2024), netball athletes (Manimmanakorn et al., 2013), trampoline athletes (Yang et al., 2022), Basketball players (Adhitya et al., 2025; Smith H. et al., 2025), and canoeists (Ugur et al., 2023). Detailed characteristics of the included studies are presented in Table 2.

**TABLE 2:** Basic characteristics of included literature.

Studies	Genders/ Subjects	Sample size		Age		Experimental group	Control group
		Experimental group	Control group	Experimental group	Control group	Interventions	Interventions
Wang et al. (2022)	M/volleyball	6	6	20.17 ± 0.75	20.83 ± 1.47	HL-BFR-RT	HL-RT
Wang et al. (2022)	M/volleyball	6	6	20.50 ± 1.38	20.83 ± 1.47	LL-BFR-RT	HL-RT
Sarfabadi et al. (2023)	M/long jump	9	8	n	n	ML-RT + LL-BFR-RT	ML-RT
Luebbers et al. (2019)	M,W/weightlifting	9	9	15.8 ± 1.2	16.6 ± 1.2	LL-BFR-RT	HL-RT
Luebbers et al. (2019)	M,W/weightlifting	9	9	15.8 ± 1.2	16.6 ± 1.2	LL-BFR-RT	LL-RT
Luebbers et al. (2014)	n/rugby players	17	14	20.3 ± 1.1	20.3 ± 1.1	HL-BFR-RT + LL-BFR-RT	HL-RT + LL-RT
Scott (2017)	M/football	10	8	19.8 ± 1.5	19.8 ± 1.5	HL-BFR-RT + LL-BFR	HL-RT + LL-RT
Hosseini et al. (2022)	n/football	10	9	15.9 ± 60.8	15.9 ± 60.8	BFR-RT	RT
Yamanaka et al. (2012)	n/football	16	16	19.2 0 ± 1.8	19.2 ± 1.8	LL-BFR-RT	LL-RT
Yang et al. (2022)	M,W/trampoline	7	8	13.9 ± 0.4	13.8 ± 0.5	LL-BFR-RT	HL-RT
Ugur et al. (2023)	n/kayak	17	16	18.59 ± 0.71	18.81 ± 1.11	LL-BFR-RT	LL-RT
Cook et al. (2014)	W/rugby players	10	10	21.8 ± 1.2	21.1 ± 1.5	ML-BFR-RT	ML-RT
Castilla-López and Romero-Franco (2023)	M/football	9	9	19.22 ± 1.69	19.22 ± 1.69	LL-RFR-RT	Interventions
Takarada et al. (2002)	W/rugby players	6	6	25.3 ± 0.8	25.4 ± 0.8	ML-BFR-RT	HL-RT
Kami̇Ş et al. (2024)	M/football	12	12	19.25 ± 0.86	19.42 ± 1.24	LL-BFR-RT	HL-RT
Adhitya et al. (2025)	M,W/basketball and rugby athletes	23	23	16.3 ± 1.4	17.1 ± 2.3	LL-BFR-RT	ML-RT
Adhitya et al. (2025)	M,W/basketball and rugby athletes	23	23	16.3 ± 1.4	16.4 ± 1.1	LL-BFR-RT	HL-RT
Smith H. et al. (2025)	M,W/basketball	8	9	21.1 ± 1.5	22.0 ± 2.1	LL-BFR-RT	LL-RT

Frequency	Duration	Resistance training exercises	Training load	BFR-RT duration, cuff position, width, pressure	Key outcome indicators	Significant differences between groups (yes or no)
3/week	8 weeks	half-squats	E:70% 1RM C:70%1RM	Throughout RT,proximal part of the thigh,7 cm, 50% arterial occlusion	Half squat 1RM, SJ	yes
3/week	8 weeks	half-squats	E:30% 1RM C:70%1RM			
2/week	6 weeks	leg press, squats	E:60%–70%1RM+20%1RM C:60%–70%1RM	Throughout RT,proximal part of the thigh, 10 cm, 60% arterial occlusion	Squat 1RM, SLJ	yes
3/week	6 weeks	squats	E:20%1RM C:65%–90%1RM	Throughout RT,proximal part of the thigh, 7.6 cm, Subjective rating of perceived exertion (RPE): 7/10	Squat 1RM	yes
3/week	6 weeks	squats	E:20%1RM C:20%1RM			
2/week	7 weeks	squats	E:≥70% 1RM+≥20%1RM C:≥70% 1RM+≥201RM	Throughout RT,proximal part of the thigh, 7.6 cm, Subjective rating of perceived exertion (RPE): 7/10	Squat 1RM, LLMH	yes
2/week	5 weeks	squats	E:60%–90%1RM+20%–30%1RM C:60%–90%1RM+20%–30%1RM	Throughout RT,proximal part of the thigh, Subjective rating of perceived exertion (RPE): 7/10	CMJ, 10 m, 20 m, 40 m, Squat 3RM	no
3/week	6 weeks	Football specialized training + lower limb strength training	E:45%–85% HRmax C:45%–85% HRmax	Throughout RT,proximal part of the thigh, 4 cm,160–210 mmHg	CMJ, Squat 1RM, 40 yard dash	yes
3/week	4 weeks	squats	E:20%1RM C:20%1RM	Throughout RT,proximal part of the thigh,5 cm,n	Squat 1RM, LLMH	yes
2/week	10 weeks	back squat, front squat	E:20%–30%1RM C:60%–85%1RM	Intermittent occlusion, proximal part of the thigh, 7.62 cm,set at 7 on the VAS scale	SJ,CMJ,LLMH	no
2/week	8 weeks	Leg Press, Leg Curl, Quadriceps Extension	E:30%1RM C:30%1RM	Throughout RT,proximal part of the thigh, 40–51 cm and 49–66 cm,180–230 mmHg	LLMH	yes
3/week	3 weeks	squats	E:70%1RM ML C:70%1RM	Intermittent occlusion, proximal part of the thigh, 10.5 cm, 180 mmHg	Squat 1RM, 40 m	yes
2/week	6 weeks	Back Squat, Single Deadlift, Barbell Hip Thrust	E:20%–35%1RM C:70%–85%1RM	Intermittent lower limbs, 7 × 82 cm,160 mmHg	Thigh girth, CMJ、30 m	no
2/week	8 weeks	knee extension	E:50%1RM C:50%1RM	Throughout RT,proximal part of the thigh, 33 mm,196 ± 5.7 mmHg	LLMH	yes
3/week	8 weeks	squat	E:bodyweight C:bodyweight	Throughout RT,proximal part of the thigh, 5 cm,60% LOP-80% LOP	CMJ, 30 m	no
2/week	8 weeks	back squats, split squats, deadlifts, and monster walks	E:30% 1RM C:70% 1RM	Throughout RT,proximal part of the thigh, 100 mm,196 ± 5.7 mmHg	Quadriceps strength	no
2/week	8 weeks	back squats, split squats, deadlifts, and monster walks	E:30% 1RM C:30% 1RM	Throughout RT,proximal part of the thigh, n,70% LAOP	Quadriceps strength	yes
3/week	4 weeks	Squats combined with speed drills (auxiliary)	E:20%–30% 1RM C:80% 1RM	Throughout RT,proximal part of the thigh, 100 mm, 60% LOP	Squat 1RM,CMJ, 5 m, 10 m, 20 m	yes

Note: M, man; W, woman; SJ, squat jump; SLJ, standing long jump; CMJ, countermovement jump; 10 m, 10-m sprint; 20 m:20-m sprint; 30 m, 30-m sprint; 40 m, 40-m sprint; LLMH, lower limb muscle hypertrophy; L, high-load; LL, low-load; ML, moderate intensity load; LL-BFR-RT, low-load blood flow restriction resistance training; RT, resistance training; LL-RT, low-load blood resistance training; n, missing data; LOP, limb occlusion pressure.

### 3.3 Risk of Bias Assessment

**FIGURE 2:**
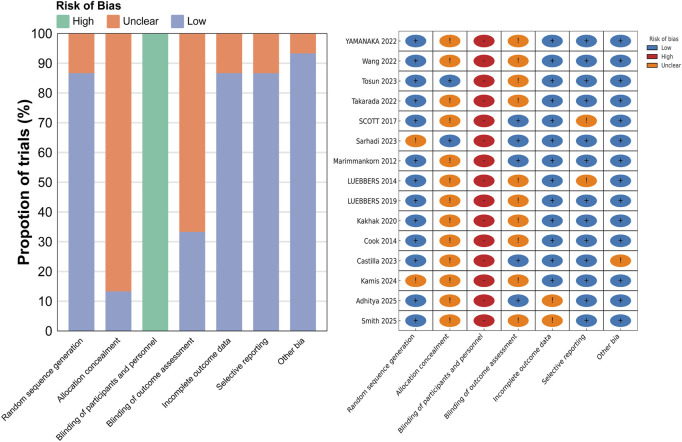
Bias risk map.

The quality assessment of the 15 included studies was conducted based on the Cochrane Risk of Bias Tool criteria, and the results are presented in Figure 2. All the included studies were randomized controlled trials. Regarding allocation concealment, 13 studies were assessed as having unclear risk, while only 2 studies were considered to have a low risk. Due to the nature of the interventions, all studies were rated as high risk for blinding of participants and personnel, as blinding was not feasible. For blinding of outcome assessment, 10 studies were assessed as having unclear risk, and 5 studies were rated as low risk. In terms of incomplete outcome data, 2 studies were assessed as having unclear risk, and 13 studies were rated as low risk. Regarding selective reporting, 2 studies were assessed as having unclear risk, while the remaining 13 studies were considered to have low risk. Regarding other biases, 1 study was assessed as having unclear risk, while the remaining studies were considered to have low risk.

### 3.4 Meta-analysis results

#### 3.4.1 Lower limb muscle hypertrophy

**FIGURE 3:**
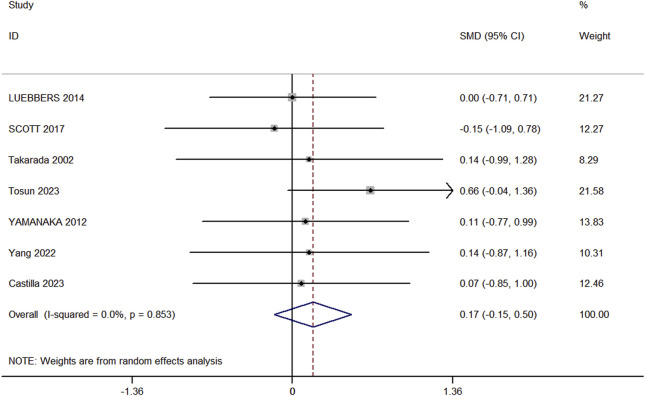
Forest plot of lower limb muscle hypertrophy in experimental and control groups.

Seven studies (comprising 7 experimental and 7 control groups, totaling 147 participants) were included in the analysis comparing the effects of RT and BFR-RT on lower limb muscle hypertrophy (Figure 3). The heterogeneity test yielded an I^2^ of 0% and a p-value of 0.853, indicating no significant heterogeneity. The meta-analysis showed a pooled effect size (ES) of 0.17 (95% CI: 0.15 to 0.50, p = 0.293), suggesting no statistically significant difference between the groups.

#### 3.4.2 Lower limb maximal strength

**FIGURE 4:**
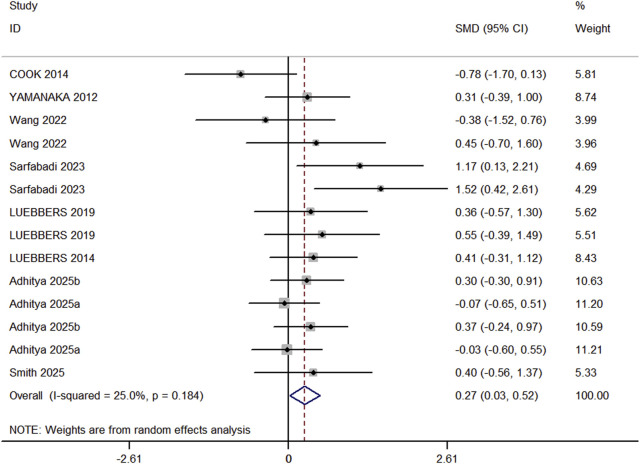
Forest plot of maximum lower limb strength between experimental group and control group.

In the included studies, 8 studies (comprising 11 experimental groups and 14 control groups, with a total of 248 participants) compared the effects of RT and BFR-RT on lower limb athletic performance in athletes (Figure 4). The heterogeneity test revealed an I^2^ value of 25% and a P value of 0.184, indicating low heterogeneity. The meta-analysis results showed a pooled effect size (ES) of 0.27 (95% CI: 0.03–0.52, p = 0.031), indicating a statistically significant difference favoring BFR-RT over RT.

#### 3.4.3 Jump performance

**FIGURE 5:**
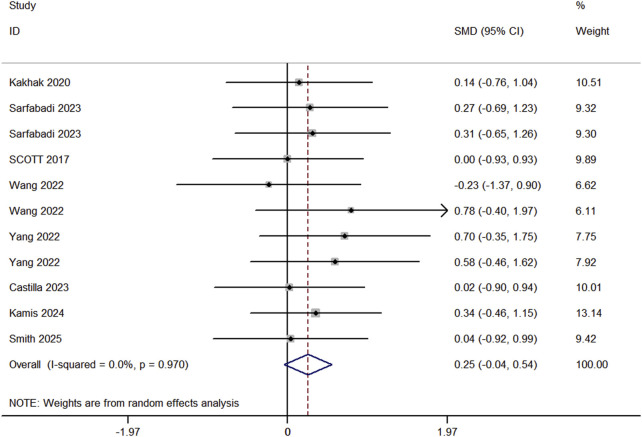
Forest plot of lower limb jumps in experimental and control groups.

Eight studies (comprising 11 experimental and 10 control groups, with a total of 207 participants) compared the effects of RT and BFR-RT on jump performance in athletes (Figure 5). The heterogeneity test indicated an I^2^ of 0% and a p-value of 0.970, showing no significant heterogeneity. The meta-analysis resulted in a pooled effect size (ES) of 0.25 (95% CI: 0.04 to 0.54, p = 0.091), with no statistically significant difference between the groups.

#### 3.4.4 Sprint speed

**FIGURE 6:**
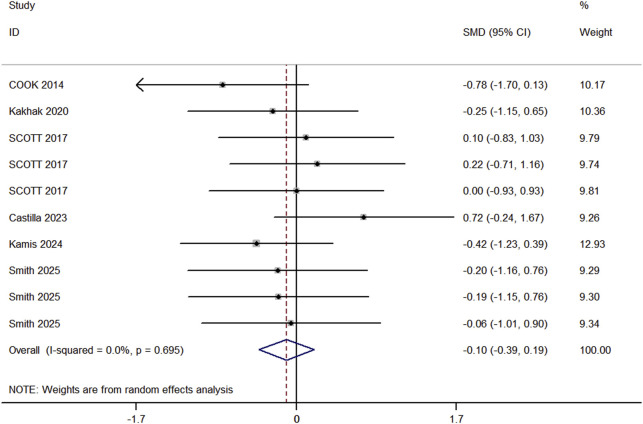
Forest plot of sprint ability between experimental group and control group.

In the included studies, 6 trials compared the sprint performance between RT and BFR-RT groups in athletes (Figure 6), comprising 9 experimental groups, 9 control groups, and a total of 201 participants. The heterogeneity test revealed I^2^ = 0% and p = 0.695, indicating substantial heterogeneity across the studies. The meta-analysis showed a pooled effect size (ES) of −0.10 (95% CI: 0.39 to 0.19, p = 0.136), with no statistically significant difference between the groups.

### 3.5 Publication bias

**FIGURE 7:**
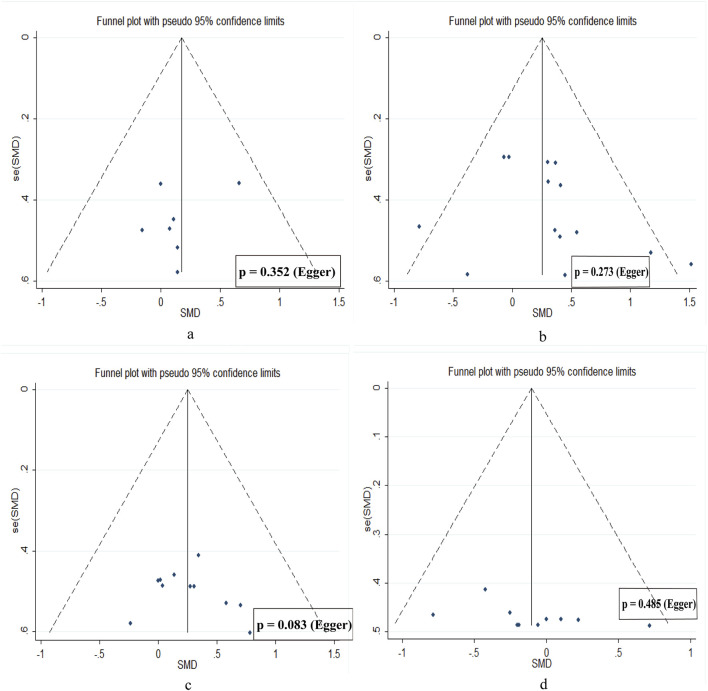
Publication bias diagram. **(a)** muscle hypertrophy. **(b)** low limb strength. **(c)** jump performance. **(d)** sprint speed.

Funnel plots for all outcomes showed approximate symmetry, and Egger’s regression tests revealed no significant publication bias, suggesting a low risk of publication bias (Figure 7).

## 4 Discussion

### 4.1 The impact of BFR-RT on lower limb muscle hypertrophy

The meta-analysis results indicate that BFR-RT is not an effective method for enhancing lower limb muscle hypertrophy in athletes when compared to RT alone. This finding is consistent with prior systematic reviews by de Ruiter et al. (2007) and Sadamoto et al. (1983), as well as many of the studies included in the present analysis (Scott et al., 2017; Yang et al., 2022; Ugur et al., 2023; Luebbers et al., 2014; Yamanaka et al., 2012), all of which reported similar results.

Individual studies agree. Laurentino et al. (2008) showed that applying an occlusion cuff during 8 weeks of heavy leg extensions did not augment quadriceps CSA gains relative to training without cuffs. Likewise, Dankel et al. (2016) found that blood flow restriction added to high-load biceps curls produced no greater muscle activation or hypertrophy than high-load exercise alone–concluding that BFR “would seem unlikely to induce greater muscle hypertrophy” under those conditions pubmed.ncbi.nlm.nih.gov. Teixeira et al. (2021) reported similar results: after 8 weeks of unilateral knee extensions, all protocols (high-load with BFR during rest, with BFR during lifting, or with no BFR) showed ∼6–7% quadriceps CSA increases with no between-group differencesjournals.lww.com. In sum, adding BFR to an already intense program did not confer any extra muscle size gain.

A likely reason is redundancy of the hypertrophic stimulus. Maximal contractions themselves generate very high intramuscular pressure, effectively occluding blood flow and causing the same hypoxic, metabolite‐rich environment that BFR aims to induce. In other words, heavy lifting “*per se*” creates near-occlusion in the muscle (Teixeira et al., 2021), so supplemental cuff pressure adds little new stress. Moreover, the once‐popular hormonal hypothesis has been largely disproven: acute surges of GH, IGF-1, testosterone, etc. from exercise have no clear link to long-term muscle growth (Lixandrão et al., 2018). Recent evidence indicates that systemic hormonal spikes do not predict how much muscle is gained–mechanical and metabolic stress appear to be the true drivers (Lixandrão et al., 2018). Thus, expecting BFR to boost growth via extra hormone release or “metabolic shock” beyond what heavy loads already provide is not supported by current knowledge.

It is worth noting that measurable muscle hypertrophy typically requires several weeks of training. For example, in Teixeira’s study, all groups achieved about 7% muscle growth after 8 weeks (Teixeira et al., 2021). Most RT studies run ≥8–12 weeks to capture muscle growth. Our meta included few very long trials, so we cannot exclude subtle effects over longer periods. Future work should employ longer interventions (e.g., ≥10–12 weeks) to determine if chronic adaptations diverge when BFR is combined with heavy lifting. Until then, the evidence suggests that for trained athletes, BFR + RT offers no hypertrophy advantage over RT alone (Teixeira et al., 2021; Lixandrão et al., 2018). Taken together, these findings suggest that in trained athletes, combining blood flow restriction with resistance training does not produce additional hypertrophic benefits compared to resistance training alone.

### 4.2 The impact of BFR-RT on lower limb maximal strength

The studies included in this analysis assessed strength by measuring the one-repetition maximum in both full and half squats, with results indicating that BFR-RT has a positive impact on lower limb strength. Additionally, the study by Yasuda et al. (2006) demonstrated that 6 weeks of blood flow restriction resistance training led to an average strength increase of 0.3 kg, further validating the effectiveness of BFR-RT in enhancing lower limb strength. Other studies have also reported significant improvements in muscular strength following BFR-RT (36), reinforcing its applicability in athletic training.

However, not all investigations concur. Gavanda et al. (2020) found no additional improvement in leg-press 1RM after 8 weeks of low-load BFR-RT (20% 1RM, 40% limb-occlusion pressure) compared with volume-matched RT in healthy males. Likewise, Lixandrão et al. (2015) observed only trivial changes in squat strength when cuff pressure was set below 40% LOP, suggesting that sub-threshold pressures may fail to elicit sufficient mechanical and metabolic stress.

Most meta-analyses and studies confirm that BFR-RT significantly enhances lower limb strength (Ugur et al., 2023; Yamanaka et al., 2012; Cook et al., 2014). The primary mechanisms underlying this effect include increased mechanical tension and metabolic stress, both of which are considered key factors in muscle adaptation and strength development (Luebbers et al., 2014; Cook et al., 2014). BFR-RT creates a hypoxic and acidic environment (Yasuda et al., 2006), promoting lactate accumulation, which in turn affects muscle contraction and sustains force output by increasing motor unit recruitment (Yamanaka et al., 2012). Additionally, the accumulation of metabolites and cellular swelling (Jessee et al., 2018; Loenneke et al., 2012) may further enhance training adaptations, while the additional recruitment of type II muscle fibers (Loenneke et al., 2015) is regarded as one of the crucial mechanisms by which BFR-RT improves strength performance. Furthermore, research suggests that BFR-RT can stimulate the secretion of anabolic hormones and accelerate the fatigue process, thereby promoting greater motor unit recruitment and overall strength gains (Yasuda et al., 2014).

From a physiological perspective, the high training frequency and appropriate loading levels in BFR-RT enhance muscle activation and increase motor unit recruitment rates during slow, high-tension contractions (Wang et al., 2022). The application of cuff pressure not only facilitates metabolite accumulation but also influences hormone secretion levels (Takano et al., 2005), thereby amplifying training adaptations. Specifically, BFR-RT elicits strength-type neural adaptations—greater motor-unit recruitment and firing rates under sustained tension—while simultaneously promoting protein synthesis, lactate accumulation, and growth-hormone release (Takano et al., 2005; Takarada et al., 2000). Notably, although BFR-RT is effective in improving strength, its adaptive mechanisms may rely more on neural adaptations rather than muscle hypertrophy (Gabriel et al., 2006).

However, several trials indicate that BFR-RT may function mainly as a complement to high-load RT rather than provide additional strength adaptations—especially when strength is evaluated via 1RM, where it often fails to surpass traditional ≥70% 1RM training (Held et al., 2020). Discrepancies across studies appear to hinge on programme and cuff-application variables. For example, Chen (Chen et al., 2022) employed twice-weekly sessions at 30% 1RM with 40% limb-occlusion pressure (LOP) and reported modest gains, whereas Hosseini Kakhak (Hosseini et al., 2022) used four sessions per week at 40% 1RM with 80% LOP and a 13 cm pneumatic cuff, yielding significantly larger improvements. Such contrasts suggest that load (<30% vs. ≥ 40% 1RM), weekly frequency (≤2 vs. ≥ 3 sessions), cuff width (≤5 cm vs. ≥ 13 cm), and—most critically—relative pressure (<50% vs. 60%–80% LOP) jointly modulate mechanical tension, metabolic stress, and thus strength adaptation. Therefore, future research should systematically manipulate these parameters to define an optimal BFR-RT prescription and to clarify its suitability across diverse athletic populations.

### 4.3 The impact of BFR-RT on jump performance

The results of the meta-analysis indicate that, compared to RT, BFR-RT is not an effective method for improving lower limb explosiveness in athletes. This finding is not entirely consistent with Xiaolin’s meta-analysis (Wang et al., 2023), which may be due to the inclusion of a “healthy population,” while our study specifically targeted athletes. This difference may have led to contrasting trends in the effects of BFR-RT on lower limb explosiveness.

First, Enhancing lower-limb explosiveness relies on rapid motor-unit firing synchrony, a high rate of force development, and efficient stretch–shortening-cycle behaviour (Enoka, 1997; Moritani, 1993). The BFR-RT interventions included in our meta-analysis mainly used slow-tempo, single-plane resistance exercises and did not incorporate plyometric or ballistic elements—stimuli that are essential for eliciting the explosive-type neural adaptations underlying vertical-jump improvements (Markovic and Mikulic, 2010; Iacono et al., 2016). These findings suggest that the specificity of the movement stimulus—rather than the blood-flow-restriction modality itself—determines whether strength-type gains translate into explosive ability.

Secondly, the impact of BFR-RT on the tendon and tendon-aponeurosis complex is also an important consideration. Existing research indicates that tendon stiffness and elasticity play a critical role in lower limb explosiveness (Maciejewska-Skrendo et al., 2020). Kubo et al.’s study found that, after 12 weeks, LL-BFR-RT did not significantly alter the tensile properties and stiffness of the tendon-aponeurosis complex. Tendon stiffness is believed to be positively correlated with explosive performance in athletes (Kubo et al., 2006; Madarame et al., 2011), especially in actions such as jumping and sprinting, where tendons contribute to force output by storing and releasing elastic energy. Therefore, the lack of significant improvement in lower limb explosiveness in this study may be related to insufficient effects of BFR-RT on tendon characteristics.

Furthermore, in Li et al. (2023) ’s research, BFR-RT was shown to indirectly promote vertical jump height by enhancing muscle metabolic adaptability and motor unit recruitment. However, the results of this study differ from LI et al.’s meta-analysis, which could be due to differences in experimental design, sample characteristics, and research conditions. Li et al. (2023)’s study may have included various types of training methods or sample groups, while our study focused specifically on resistance training, which might have had a different impact on enhancing lower limb explosiveness.

In conclusion, although BFR-RT shows potential for enhancing strength and explosiveness, its effects appear to be no greater than traditional RT. Future research could further explore the effects of BFR-RT under different conditions, such as varying athletic populations, training loads, and cycles, through subgroup analyses, to better understand its role in explosive power training.

### 4.4 The impact of BFR-RT on sprint performance

The meta-analysis results indicate that BFR-RT does not significantly improve sprint performance in athletes compared to RT alone. This finding is consistent with the majority of studies included in this analysis (Scott et al., 2017; Manimmanakorn et al., 2013; Hosseini et al., 2022; Cook et al., 2014). Research by Fostiak et al. (2022) also found that 30-m sprint performance did not improve following BFR training. The included studies evaluated sprint performance using measures such as 5-m, 10-m, 20-m, 40-m, and 40-yard sprints to comprehensively assess the impact of BFR-RT on sprint performance.

Critical factors influencing sprint performance include reaction time, technique, electromyography (EMG) activity, and neural factors. Reaction time and technique are largely influenced by genetics and training (Mero et al., 1992). In terms of other factors, EMG is often used to assess changes in motor unit recruitment, firing frequency, and synchronization (Wang et al., 2022). However, studies have shown no significant differences in EMG amplitude between BFR-RT and RT (Wang et al., 2022; Moore et al. 2004). Additionally, neural adaptations, such as increased motor unit activation and synchronization, are considered crucial for improving maximal power output (Enoka, 1997; Moritani, 1993; Fujita et al., 2008). RT research indicates that maximal voluntary contraction can increase motor unit activation (Folland and Williams, 2007). However, studies on BFR-RT have not shown significant changes in motor unit activation (Wang et al., 2022; Gabriel et al., 2006; Kubo et al., 2006; Scott et al., 2016), which may explain why BFR-RT does not significantly outperform RT in sprint performance.

It is noteworthy that one study included in our analysis found that while there was no significant difference between LL-BFR-RT and LL-RT in the 10-m sprint, LL-RT outperformed LL-BFR-RT in the 5-m sprint (Manimmanakorn et al., 2013). This suggests that BFR-RT might be more effective than RT for certain short-distance sprints. Systematic reviews by Wortman (Wortman et al., 2021) and research by Mckee et al. (2023) also pointed to the positive effects of BFR-RT on sprint performance. This might be because BFR increases metabolic stimuli during exercise, which more effectively triggers sympathetic nervous system activity, enhancing reaction speed and, consequently, displacement speed (Scott et al., 2016; Kiyohara et al., 2006).

Given these conflicting results, it is possible that BFR-RT’s effects vary with different sprint distances, and differences in BFR settings, training loads, and athlete adaptation levels may lead to varying outcomes. Therefore, more studies are needed to verify the effects of BFR-RT on specific sprint distances in different sports.

## 5 Practical applications

Our findings indicate that blood-flow-restriction resistance training (BFR-RT) is a reliable method for increasing maximal lower-limb strength. For muscle hypertrophy, explosive power, and sprint speed, BFR-RT performed comparably to traditional high-load resistance training. When size or speed is the foremost objective, programmes centred on conventional high-load work may therefore remain the first choice, with BFR-RT serving as a useful alternative whenever heavy loads are impractical—such as during rehabilitation, in-season maintenance, or deload phases. Ultimately, coaches should match the training tool to the athlete’s goals, phase of training, and tolerance for mechanical stress, integrating BFR-RT alongside traditional methods when it adds logistical or physiological value.

## 6 Limitations

Despite the systematic integration of existing research results through this meta-analysis and the evaluation of BFR-RT’s impact on athletes’ jump performance, sprint speed, lower limb maximal strength, and muscle hypertrophy, several limitations exist. First, the study only included publications in English, with only 15 studies meeting the inclusion criteria. Additionally, the majority of the studies included had intervention periods of 6–8 weeks, while some performance indicators might require longer durations to show significant effects. Future research should focus on improving sample sizes, study design, and control of external factors to provide more reliable and comprehensive evidence.

## 7 Conclusion

This meta-analysis summarizes the effects of BFR-RT on athletic performance. The results indicate that BFR-RT is superior to traditional RT in enhancing maximal lower limb strength. However, BFR-RT did not show superior effects over RT in jump performance, and no significant advantage was found in lower limb muscle hypertrophy, particularly in muscle hypertrophy and sprint speed training, where RT demonstrated more pronounced effects. Therefore, for these training objectives, RT should be prioritized.

## Abbreviations

BFRT, Blood flow restriction training; RTE, test group Resistance training; BFR-RTE, test group combining Blood Flow Restriction with Resistance Training; CMJ, Countermovement jump; 1RM, one-repetition maximum; E, test group; C, control group; SJ, Squat jump; SLJ, Standing long jump; CMJ, Countermovement jump; 10 m, 10-m sprint; 20 m, 20-m sprint; 30 m, 30-m sprint; 40 m, 40-m sprint; HL, high-load; ML, Moderate intensity load; LL, low-load; LL-BFR-RT, low-load blood flow restriction resistance training; LL-MH, Lower limb muscle hypertrophy; n, missing data. W Woman; PRISMA, Preferred Reporting Items for Systematic Reviews and Meta-Analyses; M, Man; W, Woman.
