# Correction: The effects of blood flow restriction combined with resistance training on lower limb strength, muscle hypertrophy, jumping ability, and sprint speed in athletes: a systematic review and meta-analysis

**DOI:** 10.3389/fphys.2025.1687793

**Published:** 2025-09-01

**Authors:** Beiwang Deng, Gesheng Lin, Yuer Shi, Duanying Li, Zeyun Guan, Chaoming Liang, Jian Sun

**Affiliations:** ^1^ School of Athletic Training, Guangzhou Sport University, Guangzhou, China; ^2^ Guangdong Provincial Key Laboratory of Human Sports Performance Science, Guangzhou Sport University, Guangzhou, Guangdong, China; ^3^ Department of Physical Education, Guangzhou Sport University, Guangzhou, China; ^4^ Faculty of Health Sciences and Sports, Macao Polytechnic University, Macao, China; ^5^ Badminton Technical and Tactical Analysis and Diagnostic Laboratory, Guangzhou Sport University, Guangzhou, China

**Keywords:** athletes, resistance training, lower limb strength, blood flow restriction training, athletic performance

In the published article, there were errors in the author affiliations. **Affiliation 3** was erroneously given as “School of Athletic Training, Guangzhou Sport University, Macao, China.” The correct affiliation is: “Department of Physical Education, Guangzhou Sport University, Guangzhou, China.”

**Affiliation 4** was erroneously given as “Faculty of Health Sciences and Sports, Macao Polytechnic University, Guangzhou, China. The correct affiliation is “Faculty of Health Sciences and Sports, Macao Polytechnic University, Macao, China.”

The original article has been updated.

