# Supplementary material for: The effects of blood flow restriction combined with resistance training on lower limb strength, muscle hypertrophy, jumping ability, and sprint speed in athletes: a systematic review and meta-analysis
Source: Front Physiol. 2025 Jul 29;16:1612685. doi: 10.3389/fphys.2025.1612685 (PMC12339475; doi:10.3389/fphys.2025.1612685)
Supplement: Supplementary file 1 [file DataSheet1.docx]

Supplementary Appendix

**Table of contents**

[Appendix 1: Search strategy 2](#_Toc23238)

# Appendix 1: Search strategy

**Table S1.** Search strategy of Pubmed

| **#** | **Searches** |
| --- | --- |
| 1 | (((((((Blood flow Restriction[MeSH Terms]) OR (Kaatsu[Title/Abstract])) OR (Ischemic Training[Title/Abstract])) OR (BFRT Therapy[Title/Abstract])) OR (BFRT Therapies[Title/Abstract])) OR (BFRT Therapies[Title/Abstract])) OR (Blood Flow Restriction Training[Title/Abstract])) OR (Blood Flow Restriction Exercise[Title/Abstract]) |
| 2 | ((((((resistance training[MeSH Terms]) OR (resistance exercise[Title/Abstract])) OR (Resistance[Title/Abstract])) OR (Strength Training[Title/Abstract])) OR (Strengthening Programs[Title/Abstract])) OR (Strengthening Programs[Title/Abstract]))) |
| 3 | ((((athlete[MeSH Terms]) OR (players[Title/Abstract])) OR (sportsman[Title/Abstract])) OR (sports person[Title/Abstract])) OR (sportswomen[Title/Abstract]) |
| 7 | #1 AND #2 AND #3 |

**Table S2.** Search strategy of Web of Science

| **#** | **Searches** |
| --- | --- |
| 1 | TS=("blood flow restriction" OR "kaatsu" OR "ischemic training" OR "BFRT therapy" OR "BFRT therapies" OR "blood flow restriction training" OR "blood flow restriction exercise") |
| 2 | TS=("resistance training" OR "resistance exercise" OR "resistance" OR "strength training" OR "strengthening programs") |
| 3 | TS=("athlete" OR "players" OR "sportsman" OR "sports person" OR "sportswomen") |
| 4 | #1 AND #2 AND #3 AND #4 |

**Table S3.** Search strategy of Embase

| **#** | **Searches** |
| --- | --- |
| 1 | 'blood flow restriction'/exp |
| 2 | 'kaatsu':ti,ab,kw OR 'ischemic training':ti,ab,kw OR 'BFRT therapy':ti,ab,kw OR 'BFRT therapies':ti,ab,kw OR 'blood flow restriction training':ti,ab,kw OR 'blood flow restriction exercise':ti,ab,kw |
| 3 | 'resistance training'/exp |
| 4 | 'resistance exercise':ti,ab,kw OR 'resistance':ti,ab,kw OR 'strength training':ti,ab,kw OR 'strengthening programs':ti,ab,kw |
| 5 | 'athlete'/exp |
| 6 | 'players':ti,ab,kw OR 'sportsman':ti,ab,kw OR 'sports person':ti,ab,kw OR 'sportswomen':ti,ab,kw |
| 7 | #1 OR #2 |
| 8 | #3 OR #4 |
| 9 | #5 OR #6 |
| 10 | #7 AND #8 AND #9 |

**Table S4.** Search strategy of SPORTDiscus

| **#** | **Searches** |
| --- | --- |
| 1 | SU "blood flow restriction" OR XB ("kaatsu" OR "ischemic training" OR "BFRT therapy" OR "BFRT therapies" OR "blood flow restriction training" OR "blood flow restriction exercise") |
| 2 | SU "resistance training" OR XB ("resistance exercise" OR "resistance" OR "strength training" OR "strengthening programs") |
| 3 | SU "athlete" OR XB ("players" OR "sportsman" OR "sports person" OR "sportswomen") |
| 4 | #1 AND #2 AND #3 |

**Table S5.** Search strategy of Cochrane library

| **#** | **Searches** |
| --- | --- |
| 1 | MeSH descriptor: [blood flow restriction] explode all trees |
| 2 | ('kaatsu' OR 'ischemic training' OR 'BFRT therapy' OR 'BFRT therapies' OR 'blood flow restriction training' OR 'blood flow restriction exercise'):ti,ab,kw |
| 3 | MeSH descriptor: [resistance training] explode all trees |
| 4 | ('resistance exercise' OR 'resistance' OR 'strength training' OR 'strengthening programs'):ti,ab,kw |
| 5 | MeSH descriptor: [athlete] explode all trees |
| 6 | ('players' OR 'sportsman' OR 'sports person' OR 'sportswomen'):ti,ab,kw |
| 7 | #1 OR #2 |
| 8 | #3 OR #4 |
| 9 | #5 OR #6 |
| 10 | #7 AND #8 AND #9 |
